# Evaluation of root lodging resistance during whole growth stage at the plant level in maize

**DOI:** 10.1038/s41598-022-14159-0

**Published:** 2022-06-20

**Authors:** Xiaohu Wang, Yinchang Li, Wei Han, Zhaoyu Song, Shengjian Wang, Jinzhong Yang

**Affiliations:** 1 Agronomy College, Qingdao Agricultural University, Qingdao, 266109 China; 2 Agricultural Technology Extension Center of Shandong Province, Jinan, 250013 China; 3 Qingdao Academy of Agricultural Sciences, Qingdao, 266199 China; 4 Shandong Provincial Key Laboratory of Dryland Farming Technology, Qingdao, 266109 China

**Keywords:** Computational models, Natural hazards, Environmental impact, Agroecology

## Abstract

Root lodging due to strong storm wind is a common problem in maize (*Zea mays*) production, leading to reduced crop yield and quality and harvest efficiency. Little information is available on quantifying effects of vertical leaf area distribution on root lodging in crops such as maize. Anti-lodging index of root was computed by the formula: AL_root_ = M_root_ / M_wind_, where AL denotes anti-lodging index, and M moment of force. M_root_, root failure moment of force equals to moment arm times max root side-pulling force measured in situ by means of the digital pole dynamometer, and M_wind_, wind resultant moment of force is estimated with vertical leaf area distribution and wind speed. Two maize cultivars were examined at 5 different growth stages from V8 to physiological maturity in 2019 and 2020, in Qingdao, China. Root anti-lodging index in tested cultivars fluctuated to a small extent within any year during whole growth period excluding at V8, while there was an inter-annual shift in index means (1.23 vs 0.84). Both root failure moment and wind resultant moment increased first and then decreased with the growth stage, and their influences on root anti-lodging index varied with the year. At wind grade 6, effect sizes, as contribution to root anti-lodging index, of root moment and wind moment were respectively 0.88 and 0.98. The difference in anti-lodging index between cultivars seemed to be disappearing as wind grade goes up. Root failure moment of force positively related to single root tensile resistance, root-soil ball volume, root number and total root length, whose correlation coefficient was the maximum of 0.94. Root anti-lodging index of maize proved stable from V8 on during whole growth period, and vertical leaf area distribution played a substantial role in maize root lodging in terms of wind resultant moment. Our findings provide the insights into root lodging events in crops such as maize, and would serve an approach to assessing crop root lodging resistance in breeding and cultivation programs.

## Introduction

Root lodging is a common phenomenon in maize production, which means that the plant is blown down by the strong wind, and the base of the stem is no longer perpendicular to the ground, and cannot be restored immediately after the wind stops^1^. Root lodging damages the root system, disturbs the normal canopy structure, reduces photosynthetic performance, and leads to yield reduction. Root lodging at the late growth stages results in ears touching the ground, and the sharp increase in the risk of grain rot causes a decline in quality. Needless to say, lodging also increases the difficulty of mechanical harvesting, reduces harvest efficiency. Obviously, objective and accurate identification and evaluation of root lodging resistance and understanding the evolutionary characteristics of root lodging resistance during the whole growth period are of great significance for the efficient breeding and cultivation for maize root lodging resistance.

Most studies on maize root lodging dealt with morphological and mechanical traits of roots, taking root force of resistance as indicators to lodging resistance. For example, Hébert et al. considered that root lodging rate was related to root number, root volume, root inclination angle, and root diameter from V12 to R1^2^; Fincher, Kamara, Bian et al. believed that the determination of vertical root pulling resistance at stages R1 and R2 could reflect the ability of maize roots to anchor plants^3–5^. However, there are few studies on the effects of external environmental factors, such as wind, on maize root lodging resistance. Based on the principle of internal force and external force balance of plants, Cui et al. in 2017 considered lodging as a phenomenon of directional imbalance of two kinds of forces stated previously in plants such as maize, and defined the quotient of anti-lodging index equal to the critical bending force of plants divided by the wind force on plants^6^. Based on the method above, four different plant densities of maize were compared in terms of stalk anti-lodging index, where external force dealing with vertical leaf area distribution. However, little information is available on quantifying effects of vertical leaf area distribution on root lodging in crops such as maize. On the other hand, there is a risk of root lodging in the whole growth period of maize. It has been reported that it may occur around V6 of maize^7^. Root lodging also exists before R6^8^, and it is not uncommon for root lodging to occur in R2. It is impossible to comprehensively understand maize root lodging resistance only at a certain growth stage.

The accurate evaluation of lodging resistance in maize field is helpful to the development of lodging resistant varieties, the adjustment of cultivation measures and the selection of the best planting environment. Moreover, lodging is the behavior of a plant as a whole. Therefore, while considering the external force, root anti-lodging index method will be used to study the dynamic changes of root lodging resistance of cultivars during the whole growth period in this study, aiming to quantitatively evaluate the root lodging resistance of plants during the whole growth period and verify its effectiveness, and clarify the relative importance of root anti-lodging index components and their relationship with root mechanics, morphological traits and other factors. Such a study would provide theoretical and technical support for maize root lodging resistance breeding and cultivation in the future.

## Results

### Root anti-lodging index and its components

**Figure 1:**
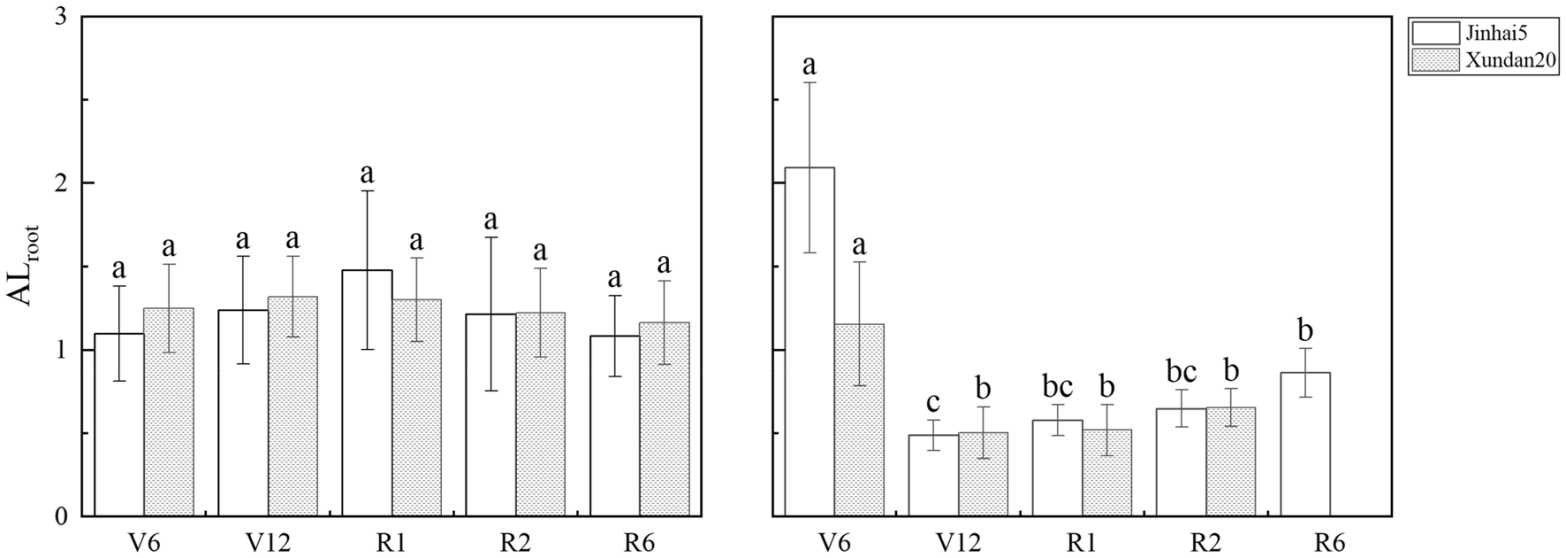
Variation trend of root anti-lodging index during whole growth period in maize.

The trend of root anti-lodging index during whole growth period is shown in Fig. 1. In 2019, root anti-lodging index of the two cultivars had the same change trend, and the value of root anti-lodging index fluctuated around 1.24, and the difference between different growth stages did not reach the statistically significant level (Table 1). In 2020, different from the results in 2019, root anti-lodging index of the two cultivars reached the maximum at the jointing stage, and root anti-lodging index of the other stages fluctuated around 0.60. The difference between different growth stages reached statistical significance. On average, root anti-lodging index in 2019 was higher than in 2020 (1.23 > 0.84). In addition, the interaction between years and varieties had a significant impact on root anti-lodging index, although not indicated in Table 1.

**Table 1:** Annual variance analysis of root anti-lodging index and its 2 components.

Year	Impact factor	AL_root_	M_root_	M_wind_
2019	Cultivar	ns	ns	*
	Growth stage	ns	**	**
	G × C	ns	**	**
2020	Cultivar	*	ns	**
	Growth stage	**	**	**
	G × C	*	ns	**

*Significant at the p < 0.05 level.

**Significant at the p < 0.01 level; ns denotes no significant difference.

**Figure 2:**
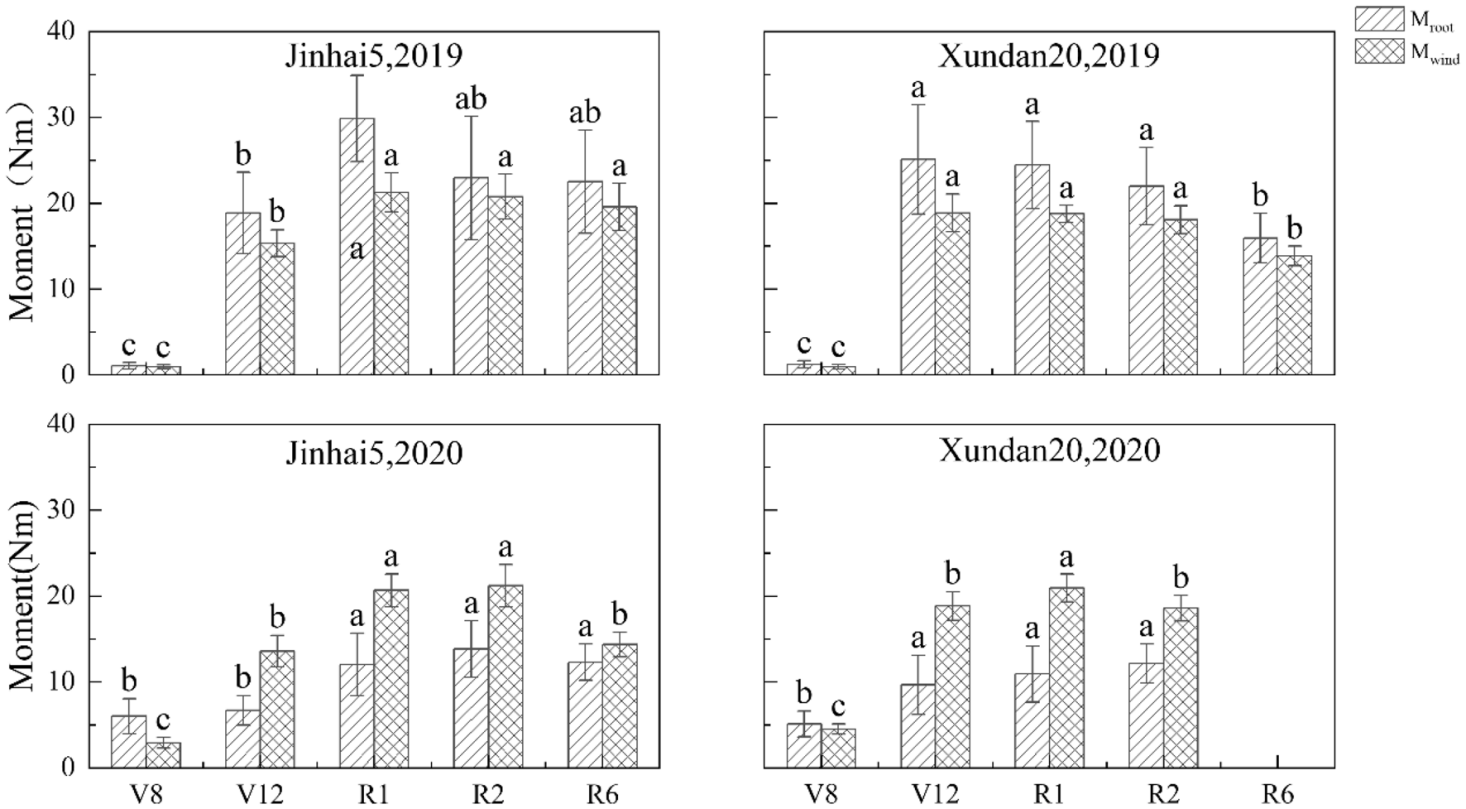
Variation trend of root anti-lodging index components during whole growth period in maize. Values in the same column followed by different lowercase letters are significantly different at the p < 0.05 level.

The changing trend of root failure moment of two cultivars was basically the same in two years, which increased first and then decreased. From V8 to R1, it basically shows an upward trend. And peaked at R1, Jinhai5 averaged 20.96 Nm, and Xundan20 averaged 17.71 Nm. From R1 to R6, basically a downward trend (Fig. 2). On average, the root failure moment in 2019 was greater than that in 2020 (18.6 > 9.88). In addition, the interaction between years and varieties had no significant effect on root failure moment, although not indicated in Table 1.

#### *Wind moment of resultant force (M*_*wind*_*)*

Wind resultant moment represents the sum of wind forces acting on plants. The greater its value is, the greater the external force is, and vice versa.

Wind moment of the two cultivars in the two years is the smallest at V8, with an average of 3.36 Nm. The basic trend of wind moment for the two cultivars was that it increased gradually from V8 to R1. And peaked at R1, with an average of 20.42 Nm. There was little difference between R1 and R2, and then decreased (Fig. 2). On average, wind moment in 2019 was roughly equal to that in 2020 (14.8 VS 15.1). In addition, the interaction between years and varieties had a significant impact on wind moment, although not indicated in Table 1.

### Effect size comparison of the influencing factors on root anti-lodging index

Effect size of root failure moment on root anti-lodging index was 0.900 at wind grade 6, roughly the same as that of wind moment, 0.968 (Table 2). This implies that the vertical leaf area distribution exerted a substantial influence with root lodging resistance in the sense of Eqs. (1) and (3).

**Table 2:** Effect size of components on root anti-lodging index at wind grade 6.

Component	ω^2^
M_root_	0.900
M_wind_	0.968

Under the factorial model, cultivar, wind grade and growth stage showed their different effects on root anti-lodging index within a wind range from grade 6 to grade 7 (Table 3). The descending order of effect size was wind grade > growth stage > cultivar, with the largest value of 0.342 for wind grade in 2019. When the wind range was enlarged from grade 6 up to grade 10, similar pattern remains of effect size, and further wind exert more influence into anti-lodging index. In 2020, there was an exception that the ranks of wind grade and growth stage were reversed.

**Table 3:** Comparison of effect size of different factors on root anti-lodging index.

Model		6 ~ 7 grade wind		6 ~ 10 grade wind	
		2019	2020	2019	2020
Factorial model	Cultivar	0.009	0.017	0.005	0.011
	Wind grade	0.342	0.145	0.718	0.492
	Growth stage	0.014	0.530	0.009	0.318
Component model	M_root_	0.947	0.181	0.781	0.150
	M_wind_	1.000	0.752	0.999	0.871

Under the component model, both root and wind moments displayed their different effect size on root anti-lodging index (Table 3). Effect size for root moment varied from 0.150 to 0.947 and for wind moment from 0.752 to 1.000. This means that wind moment imposed more influence on root anti-lodging index than root moment did regardless of years or wind ranges, and the larger the wind range was, the more profound the influence of wind moment was.

### Root anti-lodging index as a function of wind grade

**Figure 3:**
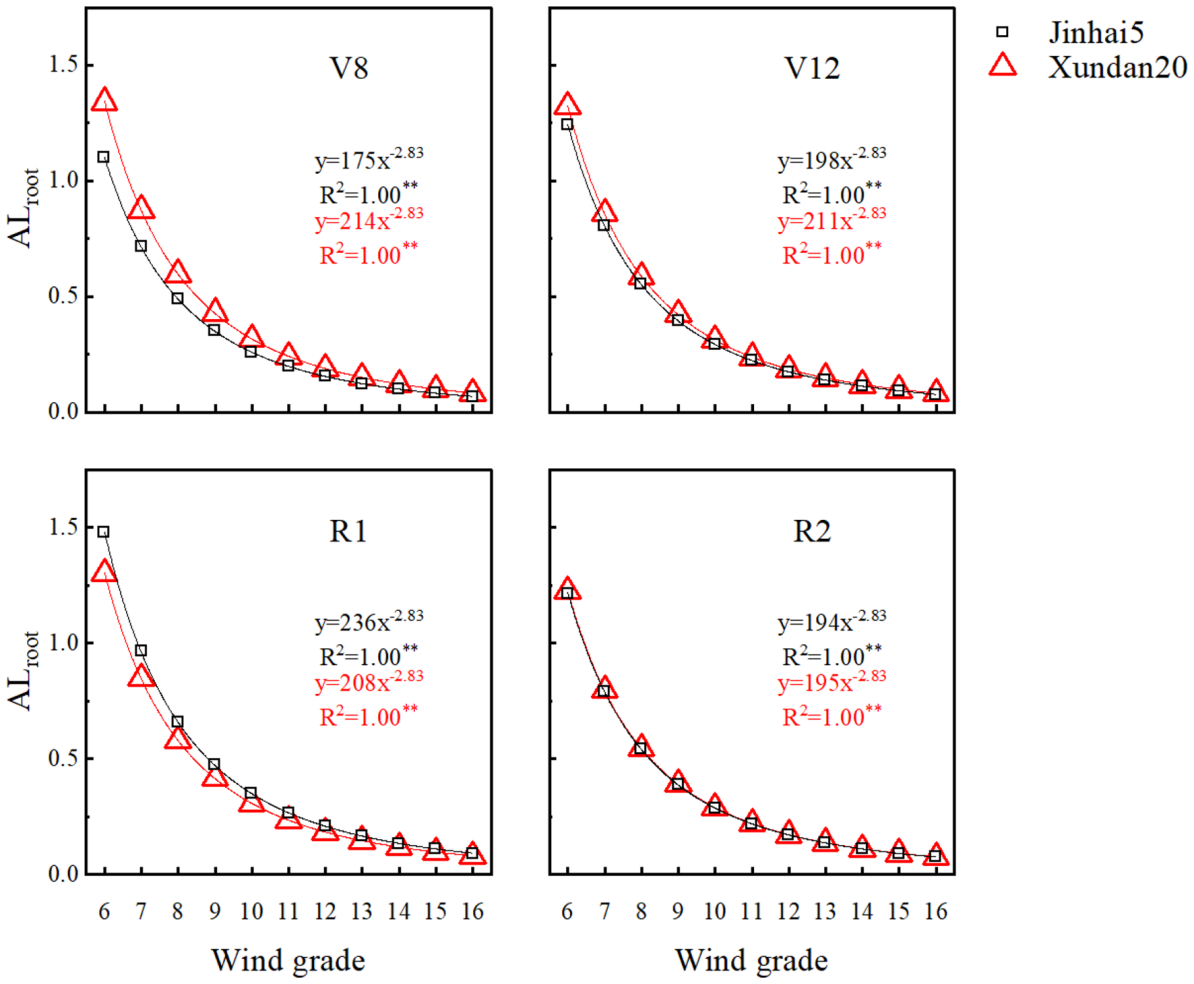
Root anti-lodging index as a function of wind grade at different growth stages for 2 tested cultivars in maize, **Statistically significant at 0.01 probability level.

Root anti-lodging index proved to be a function of wind grade that follows a negative power form regardless of grow stages or cultivars (R^2^ = 1.00, P < 0.001, Fig. 3). For a given growth stage, cultivar difference in root anti-lodging index showed to be constant in terms of ratio of Xundan20’s to Jinhai5’s. Taking V8 as an example, the ratio was 214/175 = 1.22. If one considers absolute differences between cultivars, an interesting fact appeared that cultivar difference became smaller and smaller as wind grade goes up, e.g., the difference between 1.34 for Xundan20 and 1.10 for Jinhai5 is 0.24 at wind grade 6, and the difference is 0.05 at wind grade 10. This fact implies the difference in anti-lodging index between cultivars seemed to be disappearing as wind grade goes up.

### Correlation between anti-lodging index components and other traits

**Figure 4:**
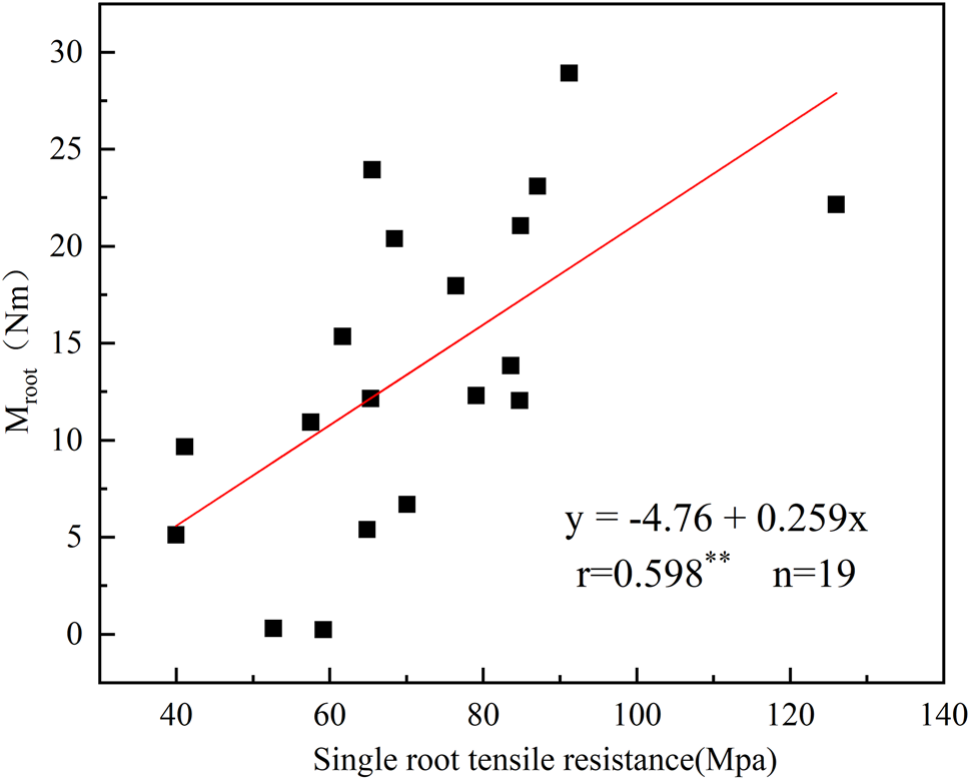
The root failure moment of force was affected by single root tensile resistance. **Statistically significant at 0.01 probability level.

The single root tensile resistance represents the ability to prevent from pulling apart of a single root. The greater its value is, the stronger the resistance is, and vice versa. Figure 4 shows the relationship between single root tensile resistance and root failure moment. Root failure moment increased linearly with increase in single root tensile resistance (r = 0.598, P < 0.001, Fig. 4).

**Figure 5:**
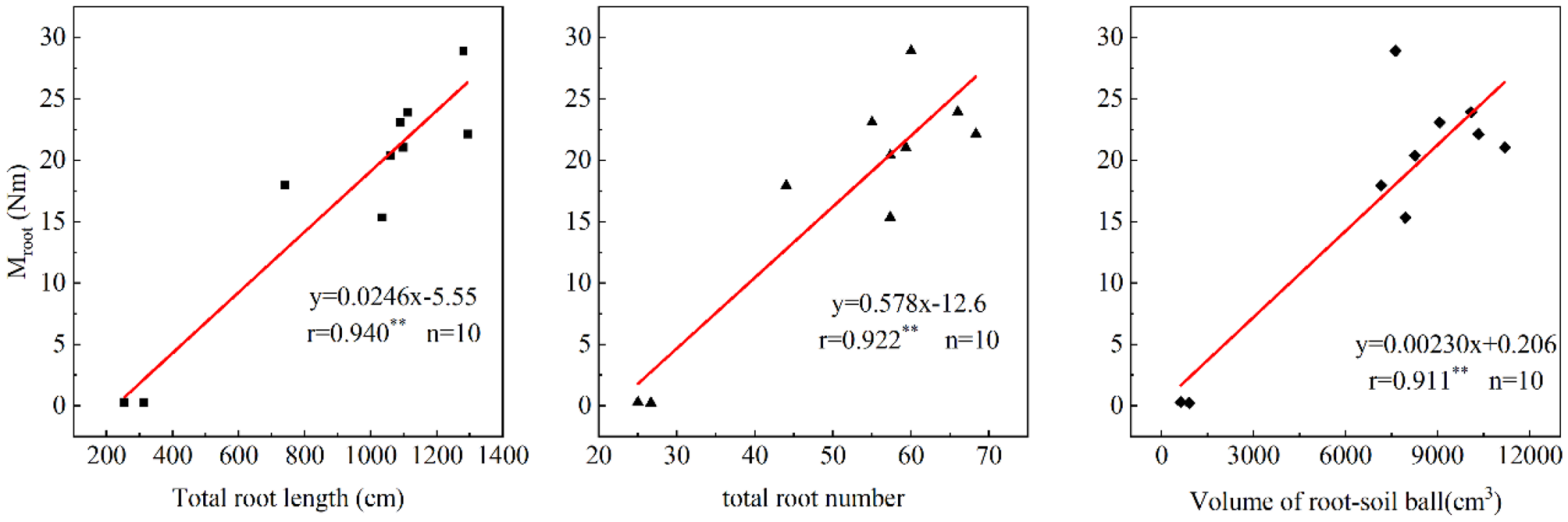
The linear relationship between root morphological traits and root failure moment. **Statistically significant at 0.01 probability level.

Root failure moment increased linearly with three root morphological traits, averaged for each of grow stages (Fig. 5). Their correlation was all at high levels of 0.940, 0.911 and 0.922 for the total length of root, the number of roots and the volume of root-soil ball respectively.

**Figure 6:**
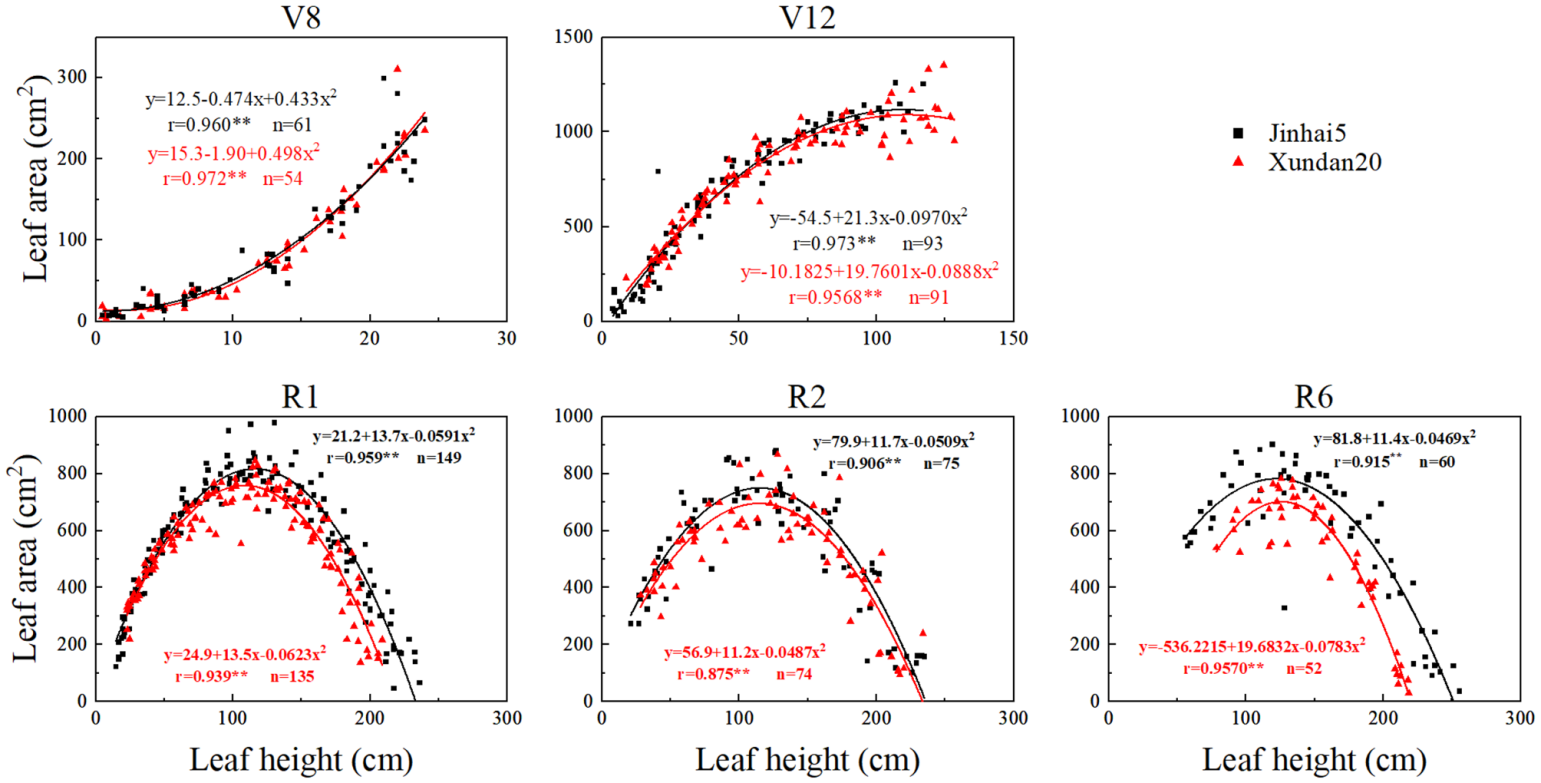
The vertical leaf area distribution during whole growth period for 2 tested cultivars of maize. **Statistically significant at 0.01 probability level.

The vertical distribution of leaf area refers to the distribution of leaf area on the main stem along with height, which decides the wind resultant moment for a given wind grade (Eq. (3)). The two tested cultivars showed the same profile of vertical leaf area distribution for the same growth stage (Fig. 6). The leaf area at V8 increased with an open upward parabolic curve, while the leaf area at V12 increased with an open downward parabolic curve. From R1 to R6, the distribution of leaf area with height was a unimodal curve. It was worth noting that the leaf area at the top height at V12 was evidently larger than that at the same height at R1 and later stages because the leaves at the top height at V12 were clustered with many unexpanded leaves in the whorl.

## Discussion

### Root anti-lodging index can objectively and quantitatively evaluate maize root lodging resistance at plant level

Root lodging is a plant behavior, which is closely related to the underground and aboveground parts of the plant body. Precise estimation of root lodging resistance must consider both the underground and aboveground parts. However, a large number of studies on maize root lodging have been regarding the resistance of roots, which are organs as parts of the plant not the plant itself, as the resistance of the whole plant, even though the literature did not necessarily clearly state this point^9–11^. Based on the comparison between the resistance force of the plant and the wind force encountered by plants, Cui et al. defined the ratio of these two forces as anti-lodging index and built a novel approach to evaluating objectively and quantitatively crop lodging resistance at the plant level^6^. At the same time, the effect of planting density on maize stem lodging resistance was successfully evaluated using this method. In this paper, this approach was used to evaluate the difference in root lodging resistance between the two maize cultivars in two years. In 2019, the difference in root anti-lodging index, root failure moment, and wind resultant moment between the two cultivars were not significant (Table 1). In 2020, there were statistically significant differences in both root anti-lodging index and wind resultant moment between the two cultivars. Root anti-lodging index of Jinhai5 was greater than that of Xundan20 (0.952 > 0.698; Fig. 1), however, the wind resultant moment of Jinhai5 was the smaller than of Xundan20 (14.5 < 15.9; Fig. 2). In addition, the vertical leaf area distribution exerted a substantial influence with root lodging resistance in the sense of Eqs. (1) and (3) (Table 2).

The mechanical characteristics of stem bending deserve attention in the study of root lodging resistance. In 2020, the root lodging rates of two maize cultivars were very different, Jinhai5 was 22%, which was evidently lower than that 72% of Xundan20 (details were not given). It was found that the difference of root anti-lodging index between the two cultivars was 0.9% (Fig. 1), which appeared to be contradictory to the degree of root lodging. The fundamental cause underlying this contradiction can be found by analyzing the mechanical process of root lodging. The process of wind blowing down maize plants actually includes two sub-processes, one is stem bending, and the other is root pulling out. These two processes offset the wind force to some extent. The root failure moment, which is the numerator of the formula for root anti-lodging index, only considers the ability of roots to resist being pulled out. This is why that the theoretical inference according to Eq. (1) is inconsistent with the actual lodging rates. This finding guarantees that the stem bending process should be paid special attention to in the following research of root lodging in maize. Therefore, to describe the natural lodging phenomenon more accurately, Eq. (1) would be rewritten with the original M_root_ replaced by M_plant_ as:

$${\text{AL}}_{{{\text{root}}}} = {\text{ M}}_{{{\text{plant}}}} /{\text{ M}}_{{{\text{wind}}}}$$

where M_plant_ denotes the bending moment of force for the plant body, including stem bending moment and root failure moment. With respect to practical operations, disuse the pair of lengthwise steel clamps when mechanical testing.

### Root lodging resistance of maize seems to be stable during the whole growth period

Many studies on maize root lodging resistance were aimed only at a given growth stage, such as R1^12^ or R2^13,14^. Although the earlier maize lodging occurs and the more serious the yield reduction is from silking stage to full maturity of maize^15^, root lodging may occur randomly at any growth stage. Therefore, studying the lodging resistance of maize in a certain stage alone will lack the overall understanding of the lodging resistance of maize, and cannot understand the dynamic change process of the lodging resistance of maize in the whole growth stage. So this study focused on the whole growth period of maize and its findings about maize root lodging resistance were more comprehensive and systematic. The results show that, the root lodging resistance dynamics of the two maize cultivars during the whole growth period showed that the fluctuation of root anti-lodging index was small and it had certain stability from stage V12 to R6 (Fig. 1). This means that in the middle and late growing season of maize, the relative size of the resistance of roots to the wind force encountered by plants remains at a relatively constant level. However, root anti-lodging index varies widely between two years (1.23 > 0.84; Fig. 1).

### Effect size can better represent the relative importance of different factors with respect to root lodging

In the analysis of many previous related studies, different factors will have the same statistical significance. For example, Zheng Yingxia^16^ found that there was a very significant positive correlation between stem breaking resistance and internode diameter, internode dry weight, dry weight per unit stem length and internode cross-sectional area. However, the significance, i.e., P values, of any effect at this time can only indicate whether an effect exists or not, but cannot provide information about the size of the effect^17^. This is to say that P’s tell nothing about the essential size of the effect so that there is no way to compare directly compare different effects. This study revealed the relative importance of factors by calculating effect size. The results showed that under the varying wind grades from 6 to 7, effect size of wind grade, growth stage and cultivar were different, the previous order of factor names being the descending order of the effects of those factors (Table 3). Effects of wind moment always were no less than that of root failure moment on root lodging in two years under a range of wind grades (Table 3). For a given wind speed, for example, wind grade 6, effect size for wind moment and root moment were similar (0.900 < 0.968, Table 2), implying that vertical leaf area distribution played a substantial role in maize root lodging.

## Conclusion

Root anti-lodging index had certain stability for the two tested cultivars from growth stage V12 to physiological maturity in maize within the same year, whereas, in which there was a large difference between two years. Root failure moment and wind resultant moment had similar effects on root anti-lodging index for a given wind grade, and the vertical leaf area distribution exerted a substantial influence with root lodging in maize. When taking into consideration wind speed variation with grades, both wind moment and wind grades showed a much higher influence on root lodging in maize. The difference in root anti-lodging index between cultivars seemed to be disappearing gradually with the increase of wind grade. The root failure moment increased with the increase in single root tensile resistance, the root-soil ball, the total length of root and the number of the root. Findings from this study provided novel insights into the root lodging resistance of maize during the whole growth period, which is worthy for reference in breeding and cultivation programs targeting at higher root lodging resistance in maize.

## Materials and methods

### Experimental design and crop management

Field experiments were conducted at Chengyang Agricultural Experimental Station, Qingdao, China (36°18′ 11"/N, 120°21′ 13"/E) in 2019 and 2020. The soil type in the field was brown loam that contained 22.76 g kg^−1^ organic matter, 82.39 mg kg^−1^ alkali-hydrolysable N, 25.10 mg kg^−1^ Olsen-P and 94.89 mg kg^−1^ exchangeable K. The test cultivars of maize were Jinhai5 with strong lodging resistance and Xundan20 with weak lodging resistance, which were repeated four times in plots laying out in randomized block designs. Plant density was 7.5 plants / m^2^ with the row spacing of 60 cm. the plot consisted of 8 rows length of 15 m. Two–three seeds per hole were manually sowed at 5 cm on 20 April 2019 and 24 April 2020, and the seedlings were thinned to the target planting density at V2, and harvested on 10 September and 14 September, respectively. Fertilization and irrigation management followed local production practices in maize.

### Sampling and measurement

Plant samples were taken at V8, V12, R1, R2 and R6. Ten typical plants of each tested cultivars were selected to be subjected to mechanical and above-ground morphological measurements at each sampling. The other three maize plants were used to measure morphological traits of roots. Xundan20 was seriously damaged due to the storm in the late stage of maize growth in 2020, resulting in the missing data for physiological maturity.

### Determination of leaf area vertical distribution

Leaf area of expanded leaves each was computed by the coefficient method: Single leaf area = length * width * 0.75. Leaf area for unexpanded leaves was estimated by the leaf weight method. Leaf area per plant was the sum of all individual green leaf areas. Leaf height is the height from the ground to the leaf collar position of maize.

### Determination of max root side-pulling resistance

**Figure 7:**
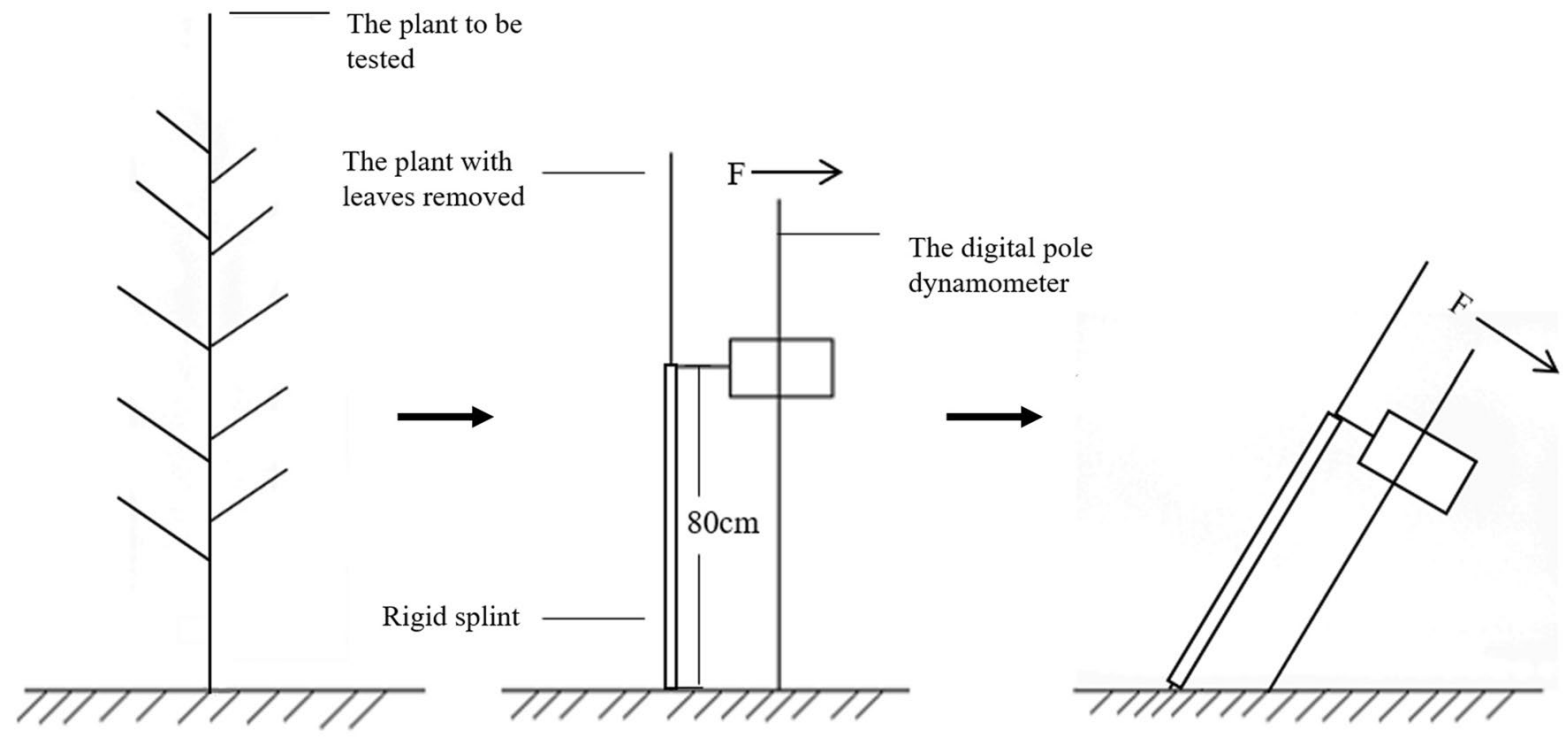
Schematic diagram for measuring max root side-pulling resistance.

Sample plants were surrounded with water-proof steel devices inserted into underground, and watered to soil moisture over saturation at one day before mechanical testing. When measured, due to the limited space, all leaves of sample plants are removed in order to improve the measurement accuracy. The defoliated stalks were immobilized by a pair of lengthwise steel clamps to prevent stalks from bending (Fig. 7). After the digital pole dynamometer^18^ with a 1.5 m long slider and a main unit was linked to the stalks at a height of 80 cm away from the ground, the operator by hand pulled at a slow and uniform speed until the roots were pulled out. Records of load force, declination angle and sensor position were automatically stored in main unit during this operation. The peak value of forces, extracted from records, was taken as the max root side-pulling resistance.

### Root anti-lodging index

Based on the method of Cui et al.^6^, the force value comparison is changed to the moment value comparison to calculate root anti-lodging index:

1 $${\text{AL}}_{root} = M_{root} / \, M_{wind} = F_{root} / \, F_{wind}$$

where *M *_*root*_ is the root failure moment, *M *_*wind*_ is the wind resultant moment. Root anti-lodging index indicates the ability of plants to resist root lodging. The larger its value is, the stronger the resistance is, and vice versa.

2 $${\text{M}}_{root} = F \, *d$$

where *F* is the max root side-pulling resistance, *d* is moment arm, i.e., the length of force arm. As a component of root anti-lodging index, the root failure moment represents the ability of the root system to resist lateral pulling. The greater its value is, the better the resistance is, and vice versa.

With the base of the stem as the fulcrum,

3 $${\text{M}}_{wind} = \sum 0.{5}CA_{i} \rho V^{2} h_{i}$$

where *C* is coefficient of air resistance, *ρ* is air mass density ,*V* is the wind speed , *A*_i_ is the area of a single leaf , *h*_i_ is the height of leaf, ∑ represents to sum up over all leaves. *C* value is set to be 0.2^19^. When encountering wind speed at grade 6 or higher, maize is more prone to lodging. Unless stated explicitly, the following analysis was limited to the upper wind speed for grade 6 wind^20^.

### Root morphological traits

The number and length of all primary nodal roots were measured. Root-soil balls each of two or three tested plants were obtained after lateral root-pulling testing. The images of the three frontal sides, 120 degrees apart from each other, of the root-soil balls were taken using a digital camera. Ball volumes were then evaluated by considering them to be rotationally symmetric. Average volumes were used for further analysis.

### Single root tensile resistance

Roots after counting the number of nodal roots were used to measure the single root tensile resistance. First, clean the dust off roots. Then, diameters of roots were determined with a vernier caliper. Single root tensile resistance was measured by HF-500 digital push–pull apparatus. Fixed the upper and lower ends of the root, then one end moved slowly and uniformly, the other end was still until the root breaks. The peak tension force displayed by the instrument was taken as the single root tensile resistance.

### Statistical analysis

Based on variance analysis, the Tukey method was used to compare the differences among means. The logarithmic transformation of variables was carried out to improve the homogeneity of error variance if appropriate.

The substantive effect or influence of various factors on the response variable can be expressed by effect size of factors, which can be calculated under the framework of variance analysis. Effect size is the proportion of the effect of a certain factor in the total effect, which is a dimensionless number^21–23^.

The formula for calculating effect size of factors is:

4 $$\omega^{2} = \frac{{df_{effect} \times \left( {MS_{effect} - MS_{error} } \right)}}{{SS_{total} + MS_{error} }}$$

where *df* is the degree of freedom, MS represents mean square.

Two conceptual models were used when dealing with effect size. One model was of components, i.e., taking the logarithm of both sides of Eq. (1):

5 $${\text{LOG}}\left( {{\text{AL}}_{{{\text{root}}}} } \right) \, = {\text{ LOG}}\left( {{\text{M}}_{{{\text{root}}}} } \right) \, + {\text{ LOG}}\left( {{\text{M}}_{{{\text{wind}}}} } \right)$$

where LOG denotes logarithmic transformation.

The other was the factorial model, i.e.,

6 $${\text{factors affecting AL}}_{{{\text{root}}}} = {\text{ wind grade }} + {\text{ cultivar }} + {\text{ growth stage}}$$

### Experimental research and field studies on plants including the collection of plant material

The authors declare that the cultivation of plants and carrying out study in Chengyang Agricultural Experimental Station complies with all relevant institutional, national and international guidelines and treaties.

### Statement of permissions and/or licenses for collection of plant or seed specimens

The authors declare that the seed specimens used in this study are publicly accessible seed materials and we were given explicit written permission to use them for this research.

## Data Availability

The datasets generated during and analyzed during the current study are available from J.Y on reasonable request.

## References

[1] Zhu, R., Ding, Y., Jiang, C. & Yang, J. Technology of preventing downfall and reducing disaster of maize in mechanical harvest era. *Mod. Agric*. 15–17 (2020).

[2] Hébert Y, Guingo E, Loudet O (2001). The response of root/shoot partitioning and root morphology to light reduction in maize genotypes. Crop Sci..

[3] Fincher RR, Darrah LL, Zuber MS (1985). Root development in maize as measured by vertical pulling resistance. Maydica.

[4] Kamara AY, Kling JG, Menkir A, Ibikunle O (2003). Association of vertical root-pulling resistance with root lodging and grain yield in selected s1 maize lines derived from a tropical low-nitrogen population. J. Agron. Crop Sci..

[5] Bian D (2016). Effects of tillage practices on root characteristics and root lodging resistance of maize. Field Crop. Res..

[6] Cui, R. et al. Lodging Index: A novel approach to evaluate lodging resistance with maize as an instance crop. *Sciencepaper Online*. (2017).

[7] Berry P (2004). Understanding and reducing lodging in cereals. Adv. Agron..

[8] Xue J (2018). Effect of lodging on maize grain losing and harvest efficiency in mechanical grain harvest. Acta Agron. Sin..

[9] Wang Q (2020). Key indicators affecting maize stalk lodging resistance of different growth periods under different sowing dates. J. Integr. Agr..

[10] Shengqun L (2019). Effects of tillage practices on root number and tensile properties in maize. Soil Crop..

[11] Bian DE, of Tillage Methods On Root Development and Root Lodging Resistance of Maize. (2013). Annual conference of Chinese crop society. Zhengzhou.

[12] Fu Z (2011). Correlation analysis of the internode number above ear and lodging resistance in Maize. J. Henan Agric. Univ..

[13] Cai H (2014). Vertical root pulling resistance and grain yield of spring maize in different planting density treatments. J. Maize Sci..

[14] Yu, D. *Study On the Effect of Brace Root Traits On Lodging Resistance and Genetic Reserch of Brace Root Traits*: Northwest A & F University, 2014.

[15] Tian, B., Yang, G., Cao, G. & Shu, H. The performent of lodging and root cause analysis for lodging resistance in crops. *Chin. Agric. Sci. Bull*. 163–167 (2006).

[16] Zheng Y (2021). Effects of planting density on lodging resistance and grain yield of spring maize stalks in Guizhou province. Acta Agron. Sin..

[17] Jinzhong Y, Xiyun S (2014). Guides to statistical techniques for single response variables in tobacco science research. Acta Tabacaria Sin..

[18] Yang, J. et al. A Lodging resistance tester for corn stem., 2017.

[19] Flesch TK, Grant RH (1991). The translation of turbulent wind energy to individual corn plant motion during senescense. Bound.-Lay. Meteorol..

[20] Zhong G (2012). Wind scale. GB/T.

[21] Olejnik SF, Algina J (2000). Measures of effect size for comparative studies: applications, interpretations, and limitations. Contemp. Educ. Psychol..

[22] Lakens D (2013). Calculating and reporting effect sizes to facilitate cumulative science: A practical primer for t-tests and ANOVAs. Front. Psychol..

[23] Ding S (2018). Study on marginal effect of edge properties of maize stalk. Shandong Agric. Sci..

